# SETD2 deficiency promotes renal fibrosis through the TGF‐β/Smad signalling pathway in the absence of VHL

**DOI:** 10.1002/ctm2.1468

**Published:** 2023-11-07

**Authors:** Changwei Liu, Li Ni, Xiaoxue Li, Hanyu Rao, Wenxin Feng, Yiwen Zhu, Wei Zhang, Chunxiao Ma, Yue Xu, Liming Gui, Ziyi Wang, Rebiguli Aji, Jin Xu, Wei‐Qiang Gao, Li Li

**Affiliations:** ^1^ State Key Laboratory of Systems Medicine for Cancer Renji‐Med X Clinical Stem Cell Research Center Ren Ji Hospital School of Medicine and School of Biomedical Engineering Shanghai Jiao Tong University Shanghai China; ^2^ School of Biomedical Engineering and Med‐X Research Institute Shanghai Jiao Tong University Shanghai China; ^3^ Department of Nursing Shanghai East Hospital Tongji University Shanghai China

**Keywords:** epigenetic regulation, renal fibrosis, SET domain‐containing 2 (SETD2), transforming growth factor‐β (TGF‐β)/Smad signalling pathway

## Abstract

**Background:**

Renal fibrosis is the final development pathway and the most common pathological manifestation of chronic kidney disease. Epigenetic alteration is a significant intrinsic factor contributing to the development of renal fibrosis. SET domain‐containing 2 (SETD2) is the sole histone H3K36 trimethyltransferase, catalysing H3K36 trimethylation. There is evidence that SETD2‐mediated epigenetic alterations are implicated in many diseases. However, it is unclear what role SETD2 plays in the development of renal fibrosis.

**Methods:**

Kidney tissues from mice as well as HK2 cells were used as research subjects. Clinical databases of patients with renal fibrosis were analysed to investigate whether SETD2 expression is reduced in the occurrence of renal fibrosis. SETD2 and Von Hippel–Lindau (VHL) double‐knockout mice were used to further investigate the role of SETD2 in renal fibrosis. Renal tubular epithelial cells isolated from mice were used for RNA sequencing and chromatin immunoprecipitation sequencing to search for molecular signalling pathways and key molecules leading to renal fibrosis in mice. Molecular and cell biology experiments were conducted to analyse and validate the role of SETD2 in the development of renal fibrosis. Finally, rescue experiments were performed to determine the molecular mechanism of SETD2 deficiency in the development of renal fibrosis.

**Results:**

SETD2 deficiency leads to severe renal fibrosis in VHL‐deficient mice. Mechanically, SETD2 maintains the transcriptional level of Smad7, a negative feedback factor of the transforming growth factor‐β (TGF‐β)/Smad signalling pathway, thereby preventing the activation of the TGF‐β/Smad signalling pathway. Deletion of SETD2 leads to reduced Smad7 expression, which results in activation of the TGF‐β/Smad signalling pathway and ultimately renal fibrosis in the absence of VHL.

**Conclusions:**

Our findings reveal the role of SETD2‐mediated H3K36me3 of Smad7 in regulating the TGF‐β/Smad signalling pathway in renal fibrogenesis and provide an innovative insight into SETD2 as a potential therapeutic target for the treatment of renal fibrosis.

## Graphical abstract

Our findings reveal the role of SETD2‐mediated H3K36me3 of Smad7 in regulating the TGF‐β/Smad signalling pathway in renal fibrogenesis. Thus, our study provides an innovative insight into SETD2 as a potential therapeutic target for the treatment of renal fibrosis

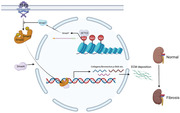

## 1 INTRODUCTION

Chronic kidney disease (CKD) is a progressive disease with no cure and affects approximately 10% of adults worldwide.^1^ Renal fibrosis is the final development pathway and the most common pathological manifestation of CKD.^2^ Characterised by deposition of extracellular matrix, such as collagen type I alpha 1 chain (COL1A1) and fibronectin (FN), renal fibrosis causes tissue scarring and ultimately leads to end‐stage renal disease.^3^ For the majority of individuals suffering from kidney failure, kidney transplantation and dialysis remain the prevailing treatment options. Nevertheless, the treatment of end‐stage renal disease poses various challenges, such as a shortage of available kidney donors, a decrease in overall quality of life and a notable mortality rate following dialysis.^4^, ^5^, ^6^, ^7^ So, it is crucial to identify and detect new potential targets to prevent renal fibrogenesis.

There is compelling literature suggesting increased expression and activation of transforming growth factor‐β (TGF‐β) in human kidney disease.^8^, ^9^ In addition, numerous animal studies have been conducted to confirm that the TGF‐β/Smad signalling pathway plays a pivotal role in driving the progression of renal fibrosis.^10^, ^11^ TGF‐β/Smad signalling pathway acts through a very typical signalling pathway that includes the phosphorylation and activation of Smad2 and Smad3 by the TGF‐β/Smad signalling pathway receptor 1 (TGFR1, also known as ALK5). Subsequently, the phosphorylated Smad2 and Smad3 bind Smad4 to form a complex that translocates into the nucleus, thereby facilitating the transcription of target genes involved in renal fibrosis.^12^ This process is inhibited by the inhibitory Smad, including Smad6 and Smad7. As a negative feedback inhibitor of the TGF‐β/Smad signalling pathway, Smad7 recruits E3 ubiquitin ligase SMAD‐ubiquitination‐regulatory factor 1 (Smurf1) and Smurf2 to degrade TGFR1.^13^ In addition, Smad7 inhibits the TGF‐β/Smad signalling pathway by competing with Smad2/3 for bounding to TGFR1 to prevent the phosphorylation of Smad2/3.^14^

An important intrinsic cause of CKD is epigenetic alteration.^15^ Epigenetics refers to changes in the transcription and expression of genes rather than changes in the genes themselves, including methylation of DNA, histone modification and non‐coding RNA.^16^ There are compelling studies suggesting that histone modifications play a crucial role in CKD and renal fibrogenesis, such as H3K9me2/3, H3K4me1/2/3, and H3K27me3.^17^, ^18^ However, the role of H3K36 methylation in renal fibrosis remains unknown. SET domain‐containing 2 (SETD2) is the sole histone H3K36 trimethyltransferase, which catalyses H3K36 trimethylation.^19^ SETD2 has been described to be implicated in a diverse array of biological processes, showing involvement in DNA repair, transcription initiation and elongation, as well as alternative splicing.^20^, ^21^, ^22^ SETD2‐mediated epigenetic alterations are implicated in many diseases, especially in renal cell carcinoma (RCC).^23^, ^24^, ^25^ Our recent studies also reported that SETD2 plays important roles in developmental areas and disease occurrence.^26^, ^27^, ^28^, ^29^, ^30^, ^31^, ^32^, ^33^, ^34^, ^35^ Notably, SETD2 deficiency accelerates the transition from polycystic kidney disease (PKD) to RCC by regulating β‐catenin activity.^26^ However, the role of SETD2 in renal fibrosis remains still unknown.

It is worth noting that Von Hippel–Lindau (VHL) is an important regulatory molecule in the pathogenesis of kidney diseases. VHL E3 ubiquitin ligase, which can recognise and ubiquitinate HIF1α and HIF2α, is associated with the development of renal fibrosis and RCC.^36^, ^37^, ^38^ Besides, mouse models combined with VHL deletion and the target molecular loss are highly favoured when studying the mechanisms underlying various kidney diseases.^39^, ^40^, ^41^

Here, we established a mouse model of renal fibrosis driven by the inactivation of SETD2 and VHL. We found that SETD2 can maintain the transcriptional level of Smad7 through H3K36me3, thus inhibiting the activation of the TGF‐β/Smad signalling pathway. SETD2 loss results in decreased Smad7 level and TGF‐β/Smad signalling pathway activation, which is predictive of fibrosis in the absence of VHL.

## 2 METHODS

### 2.1 Mouse strains

*Setd2^fl/fl^* mice were generated by Shanghai Biomodel Organism Co. The *Ksp^Cre^* mice (B6.Cg‐Tg (Cdh16‐cre) 91Igr/J) and *VHL^fl/fl^* were purchased from The Jackson Laboratory. Eight‐week‐old *Setd2^fl/fl^* mice were bred with *Ksp^Cre^* mice, resulting in offspring with the genotype *Ksp^Cre^; Setd2^fl/fl^* mice (*Setd2^−KO^*, *n* = 30). Eight‐week‐old *VHL^fl/fl^* mice were mated with *Ksp^Cre^* mice to generate *Ksp^Cre^; VHL^fl/fl^* mice (*VHL^−KO^*, *n* = 30). *Setd2^−KO^* mice were mated with *VHL^fl/fl^* mice to generate *Ksp^Cre^; VHL^fl/fl^ Setd2^fl/fl^* mice (*VHL^−KO^; Setd2^−KO^*, *n* = 30). All mice used in this study were in C57 background and were maintained in a specific pathogen‐free facility. All experimental procedures were approved by the Institutional Animal Care and Use Committee of Shanghai Jiao Tong University. The ethical number of animal experiments is 202201027.

Male and female *VHL^−KO^; Setd2^−KO^* mice, as well as *VHL^−KO^* mice, were euthanised at 15 and 30 weeks of age for subsequent experiments. Additionally, male wild‐type (WT) and *Setd2^−KO^* mice were euthanised on days 7 and 14 after unilateral ureteral obstruction (UUO) at 8 weeks of age (*n* = 10).

### 2.2 Blood urea nitrogen and creatinine test

Blood was collected from 15‐week‐old *VHL^−KO^; Setd2^−KO^* and *VHL^−KO^* mice (average weight = 30 g, *n* = 6) using the tail vein blood collection method. The serum was analysed for Blood urea nitrogen (BUN) and creatinine concentrations by Wuhan Servicebio on a Beckman Coulter AU680 analyser.

### 2.3 Isolation of primary tubular epithelial cells

To increase the percentage of Cre recombinase‐positive cells, we separated primary renal tubular epithelial cells (PTECs) from 10‐week‐old *VHL^−KO^; Setd2^−KO^* and *VHL^−KO^* mice (average weight = 25 g, *n* = 10) kidneys. Previously described procedures for isolating and identifying phenotypes were followed.^42^

### 2.4 RNA isolation and reverse transcription real‐time quantitative PCR

RNA was isolated from 10‐week‐old *VHL^−KO^; Setd2^−KO^* and *VHL^−KO^* mouse PTECs and HK2 cells using TRIzol reagent (Invitrogen) Reverse transcription real‐time quantitative PCR (RT‐qPCR) was performed using Setd2 and target gene primers with the Prime Script RT reagent kit (TaKaRa). GAPDH was used to normalise the results. Data were analysed from three independent experiments and are shown as the mean ± standard error of mean.

### 2.5 Western blot analysis and antibodies

The cells were lysed with 500 mL of Radioimmunoprecipitation assay buffer (RIPA) containing protease and phosphatase inhibitors (Millipore). Bio‐Rad's Bicinchoninic Acid (BCA) assay was used to measure protein concentrations. Proteins were separated using 6% and 10% Sodium dodecyl sulfate polyacrylamide gel electrophoresis (SDS‐PAGE) gels and then transferred to polyvinylidene fluoride membranes. The membranes were blocked with 5% bovine serum albumin (BSA) in Tris Buffered Saline (TBS) for 1 h at room temperature. They were then incubated overnight at 4°C with the primary antibody. Afterward, the membranes were washed with TBS containing 1% Tween 20 and incubated with an Horseradish Peroxidase (HRP)‐conjugated secondary antibody for 1 h at room temperature. Finally, the membranes were developed using an Enhanced chemiluminescence (ECL) reagent (Thermo). The immunoblots were quantified by Bio‐Rad Quantity One version 4.1 software. Primary antibodies against SETD2 (LS‐C332416), histone H3 (trimethylK36) (ab9050), Smad7 (ab216428) and FN (15613) were purchased from Lifespan, Abcam and Proteintech. Antibodies against histone H3 (#9715), p‐Smad3 (#9520), Smad3 (#9523), p‐Smad2 (#3108), Smad2 (#5339) and COL1A1 (#72026) were purchased from Cell Signaling Technology Inc.

### 2.6 Haematoxylin and eosin staining, immunohistochemistry and immunofluorescence

Sections of fixed tissues were stained with haematoxylin and eosin (H&E) after being fixed in 10% buffered formalin. For immunohistochemistry (IHC) staining, sections were deparaffinised, rehydrated, treated with antigen retrieval citrate buffer and quenched with 3% H_2_O_2_. Five percent BSA was used as a blocking agent for 1 h at room temperature. Afterward, primary antibodies were incubated at 4°C for 12−16 h. For immunofluorescence (IF), kidney sections were permeabilised with Triton X‐100, blocked with 5% BSA for 1 h and incubated with primary antibody at 4°C for 12−16 h. Followed by the incubation with the secondary antibodies at 37°C for 1 h. Nuclei were counterstained with 4′,6‐diamidino‐2‐phenylindole (DAPI). The primary antibodies against FN (15613), COL1A1 (#72026), p‐Smad3 (ab52903), α‐smooth muscle actin (α‐SMA) (ab184675) for IF, and Smad7 (ab216428) were purchased from Proteintech, Cell Signaling Technology Inc. and Abcam. Besides, the antibodies against α‐SMA (A17910) and SETD2 (A3194) were purchased from Abclonal. The antibodies against Tamm–Horsfall protein (THP) (sc‐271022), FITC‐LTL (FL‐1321), CD8a (14‐0808‐82) and F4/80 (17‐4801‐80) were purchased from Santa Cruz, Vector Laboratories and eBioscience.

### 2.7 RNA sequencing and analyses

RNA was extracted from 10‐week‐old *VHL^−KO^; Setd2^−KO^* and *VHL^−KO^* mice PTECs. For the construction of sequencing libraries, New England Biolabs’ Next Ultra Directional RNA Library Prep Kit for Illumina (Ipswich) was utilised. Afterward, Illumina sequencing was performed with paired‐ends 2 × 150 as the sequencing mode. Differential gene expression was analysed using the DESeq2 package. The statistically significant DE genes were obtained by a *p*‐value threshold <.05 and fold change ≥1.5. All differentially expressed mRNAs were selected for Gene Ontology (GO) analysis.

### 2.8 Chromatin immunoprecipitation‐qPCR assay

Chromatin immunoprecipitation (ChIP) assays were performed using the EZ ChIP kit (Millipore). The procedure was performed as described in the kit provided by the manufacturer. Briefly, isolated PTECs were fixed with 1% formaldehyde and broken up by sonication. Immunoprecipitation was then performed with histone H3 trimethylK36 (Abcam, ab9050). After washing and reverse cross‐linking, the precipitated DNA was amplified with primers and quantified by qPCR.

### 2.9 Masson and Sirius Red staining

Kidney samples were obtained from 15‐ and 30‐week‐old *VHL^−KO^; Setd2^−KO^* and *VHL^−KO^* mice. Kidney sections were stained with Masson's trichrome (Solar bio, G1340) and Picrosirius red (G‐CLONE, RS1220). The experiment was performed in strict accordance with the operating instructions. Measurement of the fibrotic area was quantified with ImageJ software (NIH, http://rsbweb.nih.gov/ij/).

### 2.10 SIS3 treatment

SIS3 (Selleck, S7959) was stored as a solution in Dimethyl sulfoxide (DMSO), which was used after diluting it with a medium for each assay. sh‐VHL; sg‐SETD2 HK2 cells were treated with SIS3 at 1 μM for 48 h. SIS3 (2.5 mg/kg) was administered by i.p. injection four times a week for 4 weeks in 12‐week‐old *VHL^−KO^; Setd2^−KO^* mice (average weight = 25, *n* = 5).

### 2.11 TGF‐β1 stimulation

HK2 cells were incubated in TGF‐β1 (R&D Systems, 7666‐MB) at 10 ng/mL for 12 h for Smad3 phosphorylation assessment.

### 2.12 Statistics

Statistical evaluation was conducted using Student's *t*‐test. Multiple comparisons were analysed first by one‐way analysis of variance. *p*‐Values <.05 were considered statistically significant. ^*^ *p* < .05; ^**^ *p* < .01; ^***^ *p* < .001; and ^****^ *p* < .0001.

## 3 RESULTS

### 3.1 The mRNA expression of SETD2 is positively correlated with VHL in kidney

**FIGURE 1:**
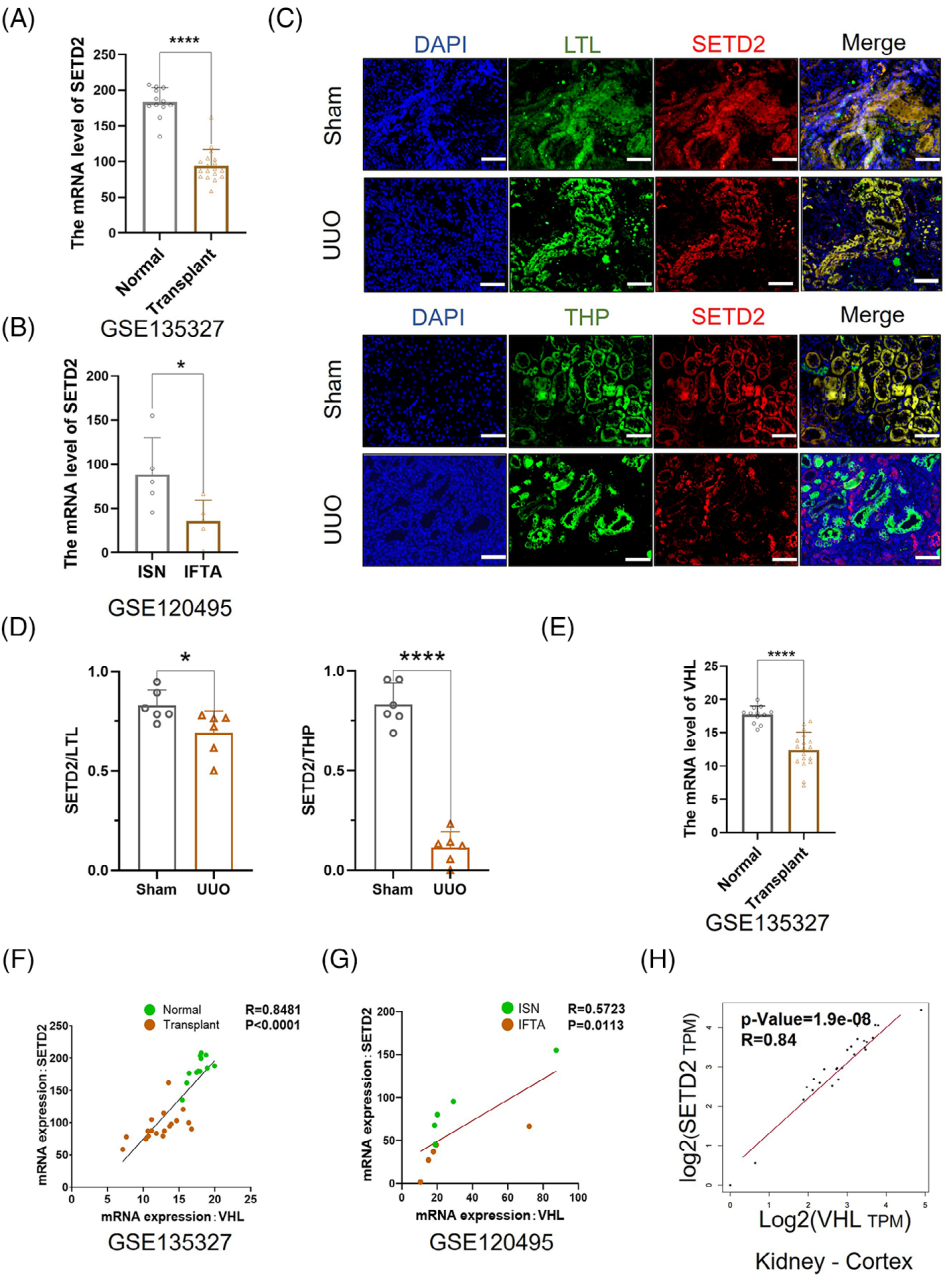
The mRNA expression of SET domain‐containing 2 (SETD2) is positively correlated with Von Hippel–Lindau (VHL) in kidney. (A) The mRNA expression level of SETD2 in normal kidneys (*n* = 12) and transplant kidneys (*n* = 18, using dataset GSE135327). (B) The mRNA expression level of SETD2 in the kidneys with interstitial nephritis (ISN) (*n* = 5) and interstitial fibrosis/tubular atrophy (IFTA) (*n* = 5, using dataset GSE120495). (C) The double immunofluorescence (IF)staining experiment results of SETD2 with Lotus tetragonolobus lectin (LTL) and Tamm–Horsfall protein (THP). Scale bars: 50 μm. (D) The statistical results of the double IF staining experiments. (E) The mRNA expression level of VHL in normal kidneys (*n* = 12) and transplant kidneys (*n* = 18, using dataset GSE135327). (F) The relation of the expression of SETD2 and VHL in normal kidneys and transplant kidneys (*n* = 5, using dataset GSE135327). (G) The relation of expressions of SETD2 and VHL in the kidneys with ISN and IFTA (using dataset GSE120495). (H) The relation of expressions of SETD2 and VHL in normal and transplant kidneys from The Cancer Genome Atlas (TCGA). The data are represented as the mean ± standard deviation (SD). ^*^ *p* < .05, ^**^ *p* < .01, ^***^ *p* < .001, ^****^ *p* < .0001.

To investigate whether SETD2 expression is altered in fibrotic kidneys, we analysed clinical data from Keenan Research Centre for Biomedical Science and found reduced expression of SETD2 in patients with renal fibrosis (Figure 1A). Considering that fibrosis is usually the result of chronic inflammation, we also analysed biopsy data from patients with nephritis and patients with renal fibrosis, and the data showed that SETD2 expression is also decreased in patients with renal fibrosis compared to patients with inflammation (Figure 1B). In addition, we generated a mouse model of UUO, commonly used in studies of renal fibrosis,^42^, ^43^, ^44^ to evaluate the expression level of SETD2 in renal fibrosis. We conducted double IF experiments using antibodies against Lotus tetragonolobus lectin (LTL) and THP, which are markers of proximal and distal tubules, respectively, to identify the tubules with SETD2 expression. As shown in Figure 1C,D, SETD2 is mainly expressed in the renal tubular epithelial cells in *WT* mice. And the expression level of SETD2 protein was decreased in the renal tubular epithelial cells in the kidney with renal fibrosis, especially in distal tubules. Meanwhile, considering that VHL deficiency can result in increased inflammation and fibrosis,^45^ we explored the relationship between SETD2 and VHL. The mRNA expression of VHL in the kidneys with renal fibrosis was also reduced compared to that in normal kidneys (Figure 1E). Meanwhile, the mRNA expression of SETD2 is positively correlated with VHL in both kidneys with renal fibrosis and normal kidneys (Figure 1F–H).

### 3.2 Loss of SETD2 is no more prone to promote renal fibrosis in the UUO model

**FIGURE 2:**
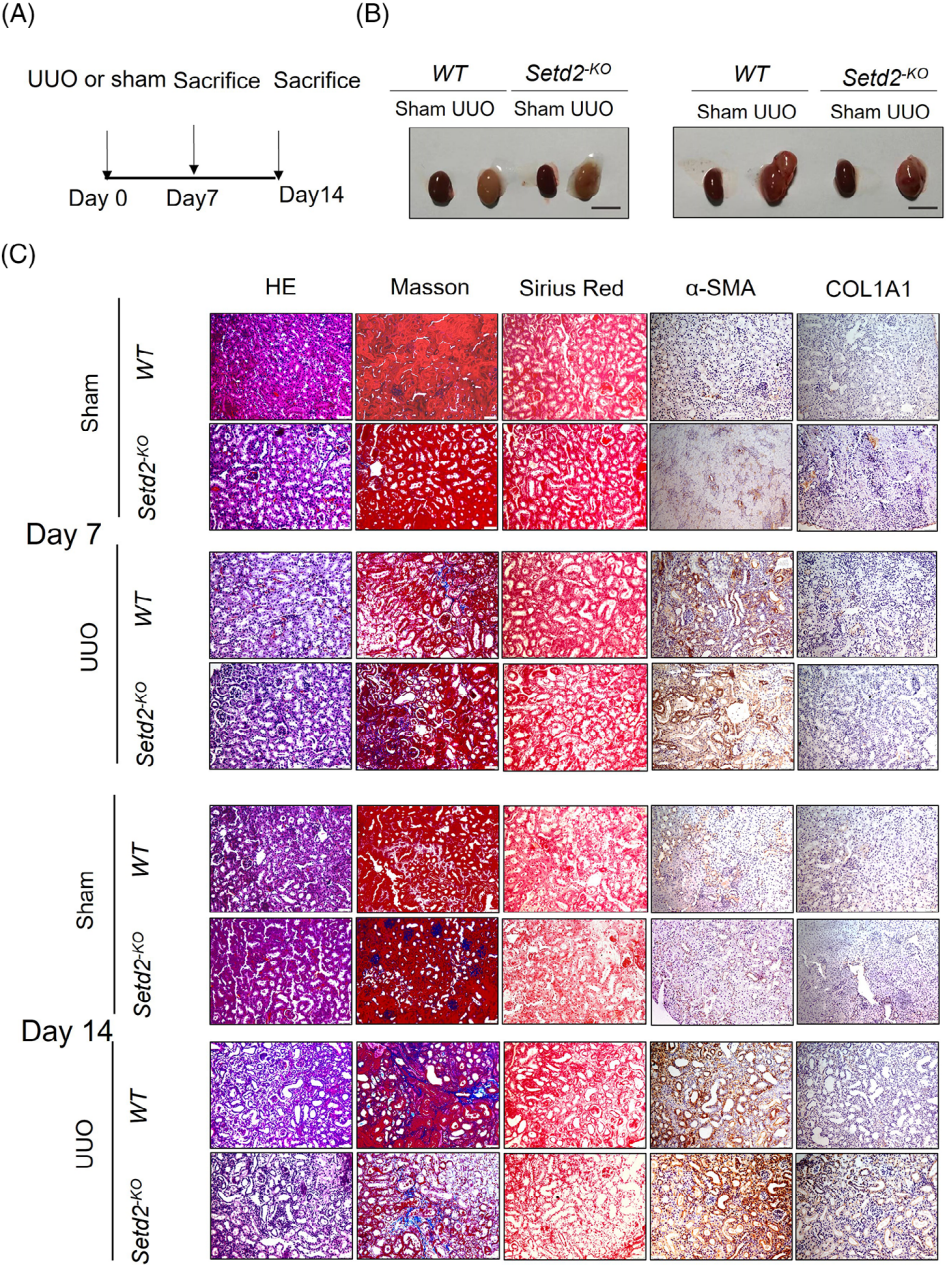
Loss of SET domain‐containing 2 (SETD2) is no more prone to promote renal fibrosis in the unilateral ureteral obstruction (UUO) model. (A) Schematic representation of generating *WT* and *Setd2^−KO^* mice UUO model (*n* = 10). (B) Kidney volumes of *WT* and *Setd2^−KO^* mice on days 7 and 14 post‐UUO (*n* = 10). Scale bars: 1 cm. (C) Haematoxylin and eosin (H&E), Masson's trichrome, Sirius Red and immunohistochemistry (IHC) staining of *WT* and *Setd2^−KO^* mice UUO model. Scale bars: 100 μm. WT, wild type.

To explore the potential function of SETD2 in renal fibrosis in vivo, we used a transgenic Ksp1.3/Cre mouse line to generate *Setd2^fl/fl^* mice and delete SETD2 in tubular epithelial cells (referred to as *Setd2^−KO^* mice).^42^ First, we generated the UUO model of *Setd2^−KO^* mice and *WT* mice (Figure 2A,B). Using Masson's trichrome and Sirius Red staining, we observed that the loss of SETD2 did not significantly increase the positive area of Masson's trichrome and Sirius Red staining compared to *WT* mice at either day 7 or 14 post‐UUO. In addition, the IHC staining results also showed that the expression levels of α‐SMA and COL1A1 remained unchanged in *Setd2^−KO^* mice compared to *WT* mice (Figure 2C). These results indicated that *Setd2^−KO^* mice were no more prone to develop renal fibrosis compared to *WT* mice under the condition of UUO.

### 3.3 SETD2 deficiency resulted in severe renal fibrosis in VHL‐deficient mice

To further investigate whether SETD2 contributes to renal fibrosis, we generated *Ksp^Cre^; VHL^fl/fl^* mice (hereafter referred to as *VHL^−KO^* mice) and *Ksp^Cre^; VHL^fl/fl^; Setd2^fl/fl^* mice (hereafter referred to as *VHL^−KO^; Setd2^−KO^* mice) (Figure 3A). Based on the experimental results obtained from Western blotting (WB), IHC and RT‐qPCR, we observed a downregulation of VHL, SETD2 and H3K36me3 expression levels in the renal tubules (Figure 3B–D). Observing the mouse kidneys, we found that the kidneys appeared to be crumpled and smaller in *VHL^−KO^; Setd2^−KO^* mice than in *VHL^−KO^* mice (Figure 3E). To observe the morphologic alteration, we performed H&E, Masson's trichrome and Sirius Red staining, and the results showed that *VHL^−KO^; Setd2^−KO^* mice exhibited a loss of normal renal tubular structure and numerous cysts. Additionally, these mice displayed characteristic features of renal fibrosis (Figure 3F,G). Furthermore, we performed RT‐qPCR and found a significant upregulation in the expression of inflammatory cytokines and chemokines in *VHL^−KO^; Setd2^−KO^* mice compared with *VHL^−KO^* mice, including Interleukin‐1α (IL‐1α), Interleukin‐6 (IL‐6), Chemokine (C‐X‐C motif) ligand 1 (CXCL1) and Chemokine (C‐C motif) ligand2 (CCL2). Similarly, IF staining revealed an increased infiltration of macrophages and CD8^+^ T cells in *VHL^−KO^; Setd2^−KO^* mice compared with *VHL^−KO^* mice (Figure 3H,I). Meanwhile, the expression levels of COL1A1, α‐SMA and FN in *VHL^−KO^; Setd2^−KO^* mice were much higher than that in *VHL^−KO^* mice, suggesting that SETD2 deficiency leads to renal fibrosis (Figure 3J,K). Moreover, in line with the morphologic alteration, BUN and creatinine levels were reduced in the blood of *VHL^−KO^; Setd2^−KO^* mice compared to the control group (Figure 3L). Together, these results indicated that SETD2 deficiency resulted in severe renal fibrosis in VHL‐deficient mice.

FIGURE 3SET domain‐containing 2 (SETD2) deficiency resulted in severe renal fibrosis in Von Hippel–Lindau (VHL)‐deficient mice. (A) Schematic representation of generating the *VHL^−KO^* and *VHL^−KO^; Setd2^−KO^* mouse model (*n* = 30). (B) Immunohistochemistry (IHC) staining of VHL and H3K36me3 in *WT*, *VHL^−KO^* and *VHL^−KO^; Setd2^−KO^* mice (*n* = 8). Scale bars: 25 μm. (C) Western blotting (WB) analysis of the expression of SETD2 in *WT* and *VHL^−KO^; Setd2^−KO^* mice (*n* = 8). (D) The mRNA level of SETD2 in *VHL^−KO^* and *VHL^−KO^; Setd2^−KO^* mice. The mRNA level of VHL in *WT* and SETD2^–KO^ mice (*n* = 8). (E) Kidney volumes of *VHL^−KO^* and *VHL^−KO^; Setd2^−KO^* mice (*n* = 8). Scale bars: 1 cm. (F) Haematoxylin and eosin (H&E), Masson's trichrome and Sirius Red staining of 15‐ and 30‐week‐old *WT*, *Setd2^−KO^*, *VHL^−KO^* and *VHL^−KO^; Setd2^–KO^* mice. Scale bars: 50 μm (left), 25 μm (right). (G) The statistical results of 15‐ and 30‐week‐old *VHL^−KO^* and *VHL^−KO^; Setd2^−KO^* mice (*n* = 10). (H) The RT‐qPCR results of the inflammatory cytokines and chemokines in *VHL^−KO^* and *VHL^−KO^; Setd2^−KO^* mice. (I) Testing the infiltration of macrophages and CD8^+^ T cells in 15‐week‐old *VHL^−KO^* and *VHL^−KO^; Setd2^−KO^* mice by using the antibodies against F4/80 and CD8α. Scale bars: 100 μm (left), 50 μm (right). (J) Immunohistochemistry (IHC) and immunofluorescence (IF) results of COL1A1 and α‐smooth muscle actin (α‐SMA) in 15‐week‐old *VHL^−KO^* mice and *VHL^−KO^; Setd2^−KO^* mice (*n* = 10). Scale bars: 100 μm. (K) The mRNA levels of collagen type I alpha 1 chain (COL1A1), fibronectin (FN) and α‐SMA in 15‐week‐old *VHL^−KO^* mice and *VHL^−KO^; Setd2^−KO^* mice. (L) BUN and creatinine levels in 15‐week‐old *WT*, *Setd2^−KO^*, *VHL^−KO^* and *VHL^−KO^; Setd2^−KO^* mice (*n* = 6 mice per group). The data are represented as the mean ± standard deviation (SD). ^*^ *p* < .05, ^**^ *p* < .01, ^***^ *p* < .001, ^****^ *p* < .0001. WT, wild type.

**Figure:**
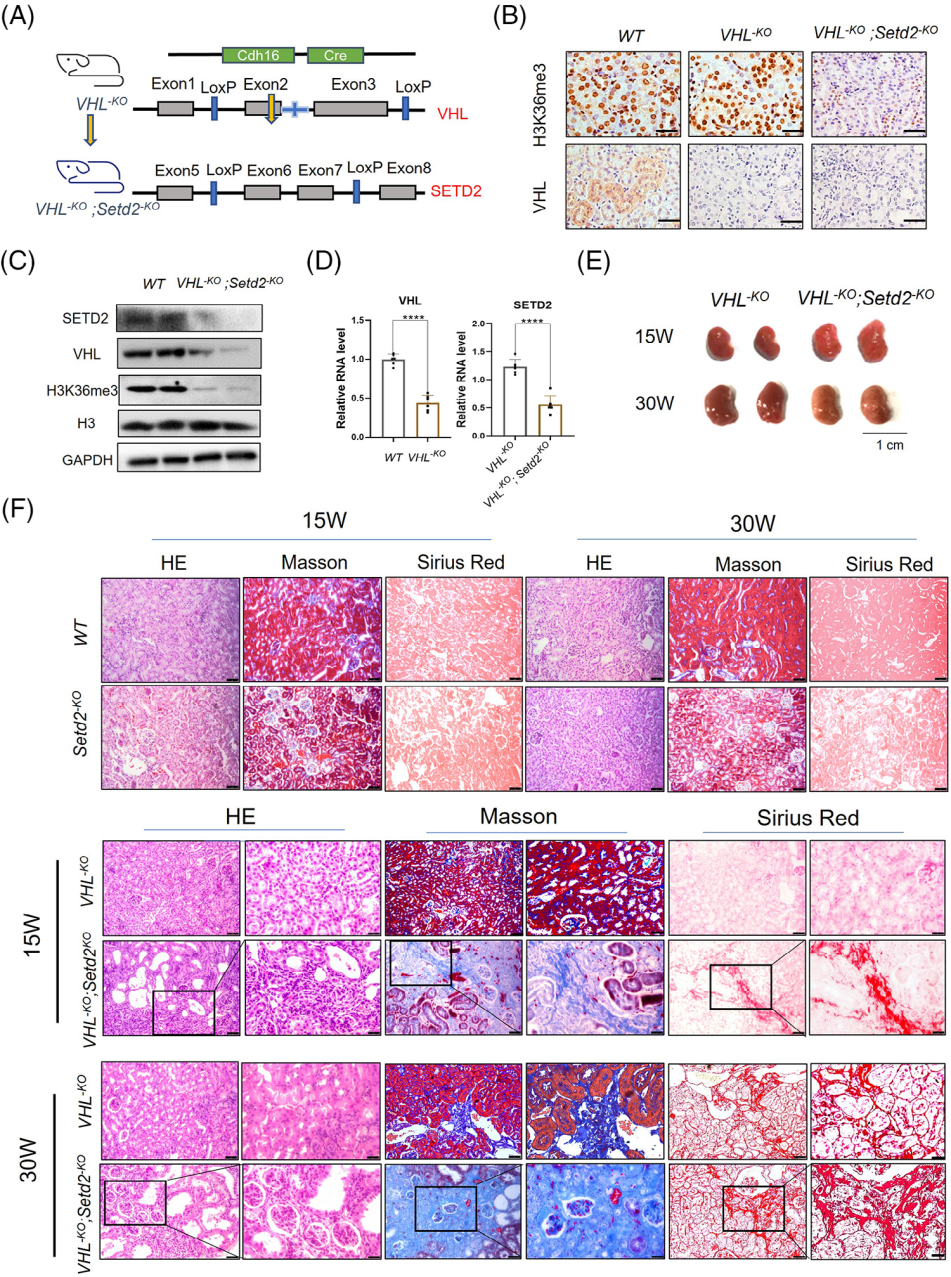

**Figure:**
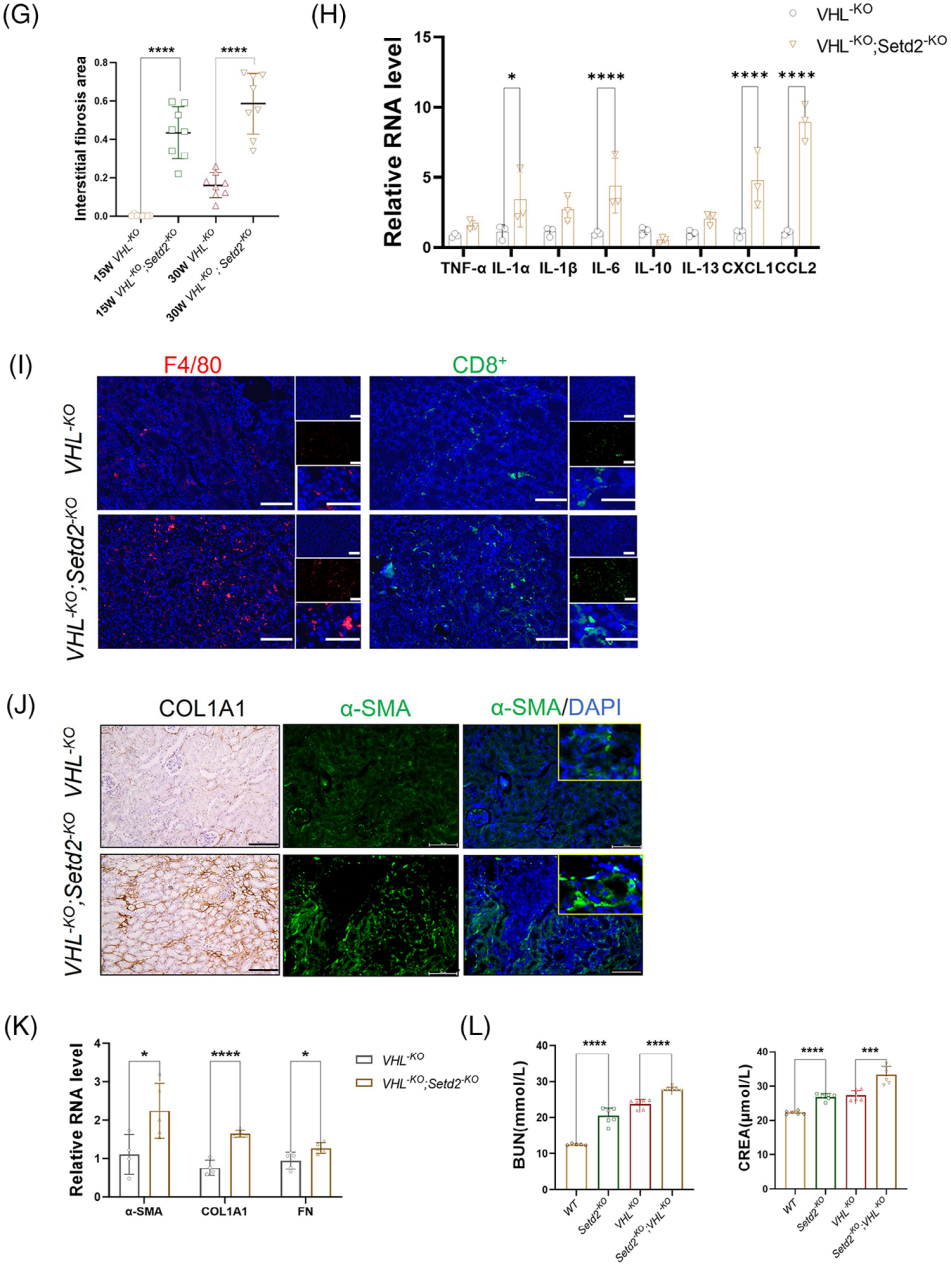

### 3.4 The inactivation of SETD2 caused hyperactive TGF‐β/Smad signalling pathway in VHL‐deficient renal tubular epithelial cells

**FIGURE 4:**
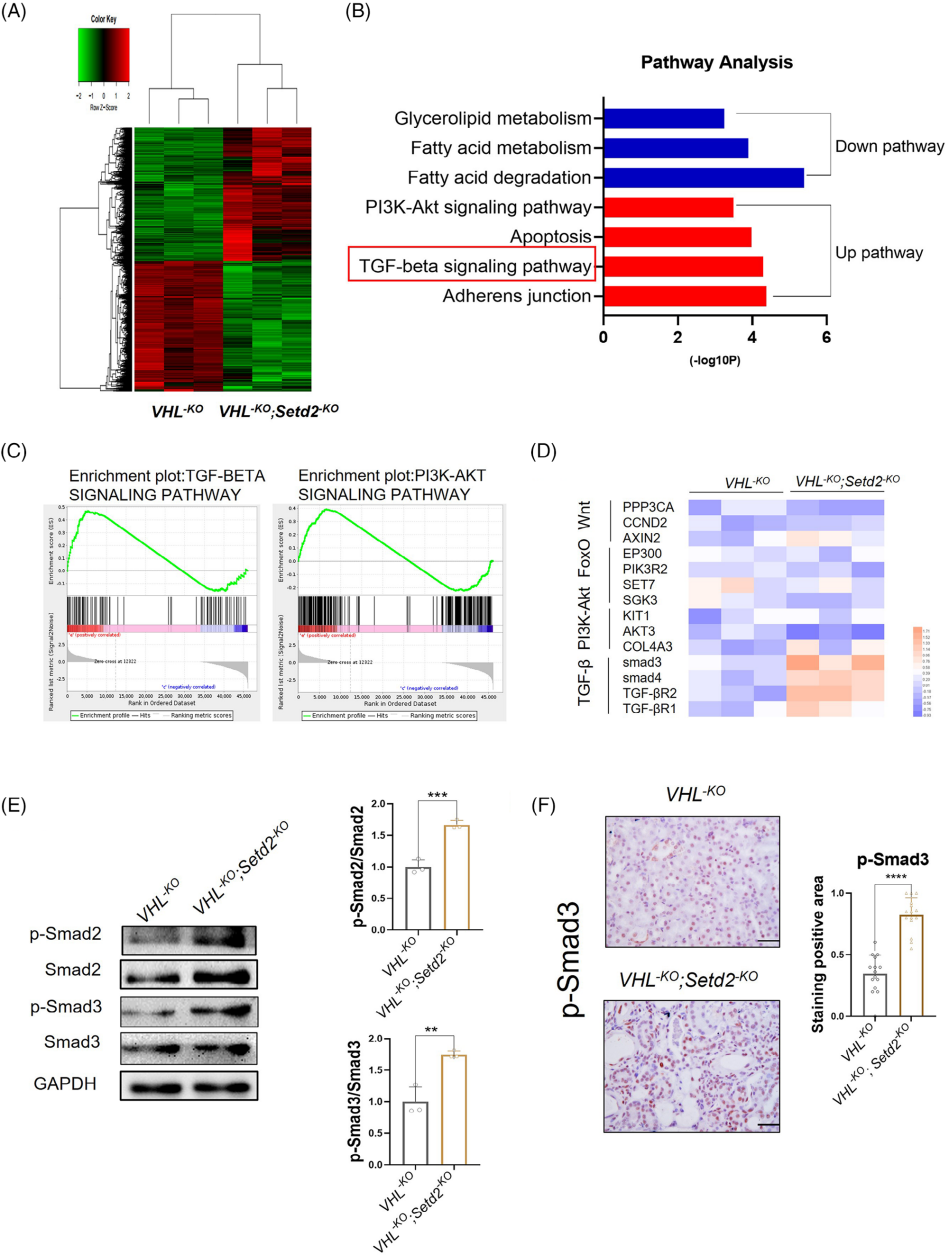
The inactivation of SET domain‐containing 2 (SETD2) caused hyperactive transforming growth factor‐β (TGF‐β)/Smad signalling pathway in Von Hippel–Lindau (VHL)‐deficient renal tubular epithelial cells. (A) Heatmap of genes with significantly different expression in freshly isolated renal tubular epithelial cells based on unsupervised hierarchical agglomerative clustering (*n* = 3 mice per group). (B) Pathway analysis of differentially expressed genes belonging to signalling pathways associated with SETD2 deletion. (C) Gene set enrichment analysis (GSEA) enrichment plots of differentially expressed genes associated with SETD2 deletion in VHL‐deficient tissue. (D) RT‐qPCR analysis of Wnt signalling pathway, TGF‐β signalling pathway, FoxO signalling pathway and PI3K signalling pathway (*n* = 3 mice per group). (E) The protein levels of p‐Smad2 and p‐Smad3 in *VHL^−KO^* and *VHL^−KO^; Setd2^−KO^* mice. (F) Immunohistochemistry (IHC) staining reveals the positive rate of p‐Smad3 in *VHL^−KO^* and *VHL^−KO^; Setd2^−KO^* mice (*n* = 8). Scale bars: 25 μm. The data are represented as the mean ± standard deviation (SD). ^*^ *p* < .05, ^**^ *p* < .01, ^***^ *p* < .001, ^****^ *p* < .0001.

To explore the mechanisms by which SETD2 deficiency promotes renal fibrosis in VHL‐deficient renal tubular epithelial cells, we performed RNA sequencing using PTECs freshly isolated from 10‐week‐old *VHL^−KO^* and *VHL^−KO^; Setd2^−KO^* mice. The overall transcriptome of *VHL^−KO^; Setd2^−KO^* PTECs was significantly altered compared to *VHL^−KO^* PTECs. A total of 2659 genes were upregulated and 2617 genes downregulated with a fold change ≥1.5 (Figure 4A). GO and pathway analysis presented that SETD2 inactivation significantly enriched lots of genes associated with pathways associated with fibrogenesis. In addition, GO analysis showed that several pathways were upregulated in *VHL^−KO^; Setd2^−KO^* mice compared with *VHL^−KO^* mice, including the TGF‐β/Smad signalling pathway and PI3K–Akt signalling pathway (Figure 4B). We conducted gene set enrichment analysis to gain a better understanding of the signalling pathways mediated by SETD2. The data showed that TGF‐β/Smad signalling pathway‐associated genes were enriched in *VHL^−KO^; Setd2^−KO^* PTECs. To verify the results of the pathway analysis and explore the pivotal pathway in fibrogenesis, we used RT‐qPCR to detect the expression levels of related pathways in more samples, including the TGF‐β/Smad signalling pathway, PI3K–Akt signalling pathway, FoxO signalling pathway and Wnt signalling pathway. And the results showed that only the TGF‐β/Smad signalling pathway was indeed significantly activated in *VHL^−KO^; Setd2^−KO^* mice (Figure 4D). TGF‐β/Smad signalling pathway plays an important role in renal fibrogenesis and a wide range of studies have established the TGF‐β/Smad signalling pathway as the paramount pathogenic factor that drives fibrosis. The activated Smad2 and Smad3 have been verified in *VHL^−KO^; Setd2^−KO^* mice by WB (Figure 4E). Furthermore, the data of IHC also showed that the positive rate of p‐Smad3 was much higher in *VHL^−KO^; Setd2^−KO^* mice than that in *VHL^−KO^* mice (Figure 4F). These results indicated that SETD2 can inhibit the TGF‐β/Smad signalling pathway transcriptional activity and SETD2 loss leads to p‐Smad2 and p‐Smad3 activation, which activates TGF‐β/Smad signalling in VHL‐deficient kidneys.

### 3.5 SETD2 inhibits TGF‐β/Smad signalling activation by regulating Smad7 expression

**FIGURE 5:**
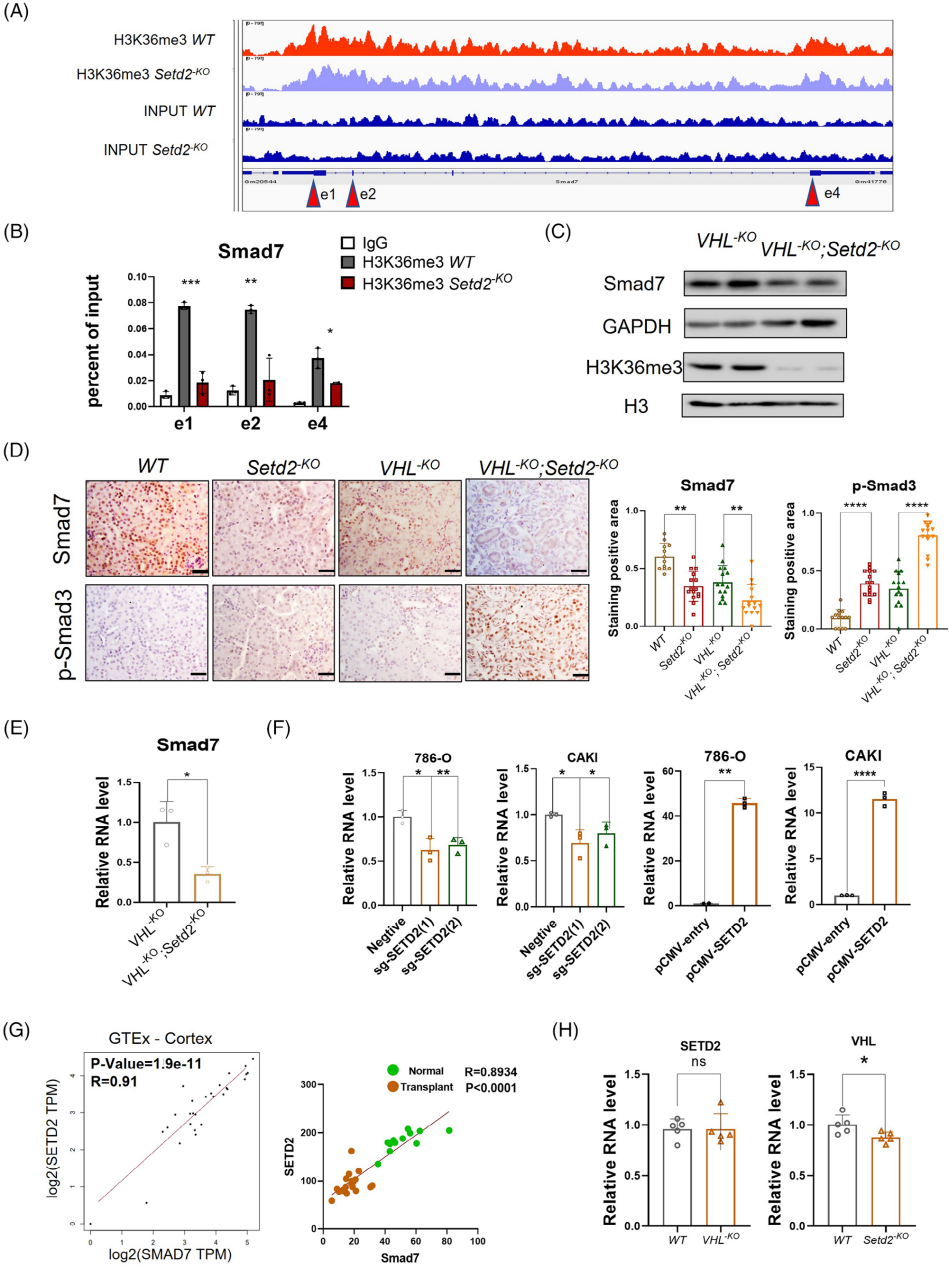
SET domain‐containing 2 (SETD2) inhibits transforming growth factor‐β (TGF‐β)/Smad signalling activation by regulating Smad7 expression. (A) Snapshot of H3K36me3 chromatin immunoprecipitation sequencing (ChIP‐Seq) signals at the Smad7 gene loci in primary tubular epithelial cells (PTECs). (B) ChIP‐qPCR analysis of H3K36me3 occupancy to gene body‐retained locus using immunoglobulin G (IgG) as the control (*n* = 3). (C) Western blot analysis of the Smad7 protein level in *VHL^−KO^* and *VHL^−KO^; Setd2^−KO^* mice. (D) Immunohistochemistry (IHC) staining and statistics of Smad7 and p‐Smad3 in *WT*, *Setd2^−KO^*, *VHL^−KO^* and *VHL^−KO^; Setd2^−KO^* mice (*n* = 8). Scale bars: 50 μm. (E) The mRNA expression levels of Smad7 in *VHL^−KO^; Setd2^−KO^* mice and *VHL^−KO^* mice (*n* = 8). (F) Changes in Smad7 mRNA expression in 786‐O and CAKI cells after SETD2 knockdown or overexpression (*n* = 3). (G) Correlation between SETD2 and Smad7 expression levels in normal kidney tissues and GSE135327. Statistical significance was determined using the Pearson correlation coefficient. (H) The SETD2 expression level in 10‐week‐old *WT* and *VHL^−KO^* mice and Von Hippel–Lindau (VHL) expression in 10‐week‐old *WT* and *Setd2^−KO^* mice. The data are represented as the mean ± standard deviation (SD). ^*^ *p* < .05, ^**^ *p* < .01, ^***^ *p* < .001, ^****^ *p* < .0001. WT, wild type.

To explore the potential mechanisms of SETD2‐dependent epigenetic alterations and to identify genes associated with SETD2 and H3K36me3 on a genome‐wide scale in the kidney, we referred to the ChIP sequencing data as described to explore possible SETD2/H3K36me3 targets.^26^ Considering that SETD2 can facilitate gene transcription, we investigated the negative regulators of the TGF‐β/Smad signalling pathway and we found that the H3K36me3 antibody occupies the Smad7 transcription start region and exon region (Figure 5A). To demonstrate the involvement of SETD2 and H3K36me3 histone marker in Smad7 transcription, we verified the occupancy of H3K36me3 in the Smad7 transcribed region using ChIP‐qPCR (Figure 5B). These results indicated that SETD2 deletion eliminates H3K36me3 modification in the transcriptional region Smad7. In addition, Smad7 protein expression was also reduced in *VHL^−KO^; Setd2^−KO^* mice compared with *VHL^−KO^* mice (Figure 5C). Furthermore, IHC and RT‐qPCR results also showed that SETD2 loss resulted in the downregulation of Smad7 expression in *VHL^−KO^; Setd2^−KO^* mice PTECs (Figure 5D,E). To better verify the fact that SETD2 can regulate Smad7 transcription in vitro, we utilised renal cell lines and demonstrated that SETD2 depletion leads to a reduction in Smad7 transcript level, while overexpression of SETD2 leads to a large increase in Smad7 expression (Figure 5F). In addition, clinical data also showed a positive correlation between SETD2 and Smad7 expression (Figure 5G). Together, these results showed that SETD2 inhibits TGF‐β/Smad signalling activation by regulating Smad7 expression. Notably, RT‐qPCR analysis showed that VHL mRNA expression level was reduced by SETD2 deficiency in the PETCs from 10‐week‐old mice (Figure 5H). This result suggested that SETD2 loss also partially affects the inhibitory function of VHL on the TGF‐β signalling pathway. Furthermore, in *VHL^−KO^; Setd2^−KO^* mice PTECs, VHL is deleted in the renal tubules, leading to activation of the TGF‐β/Smad signalling pathway.

### 3.6 SETD2 deletion leads to fibrosis through activation of the TGF‐β/Smad signalling pathway in vitro

**FIGURE 6:**
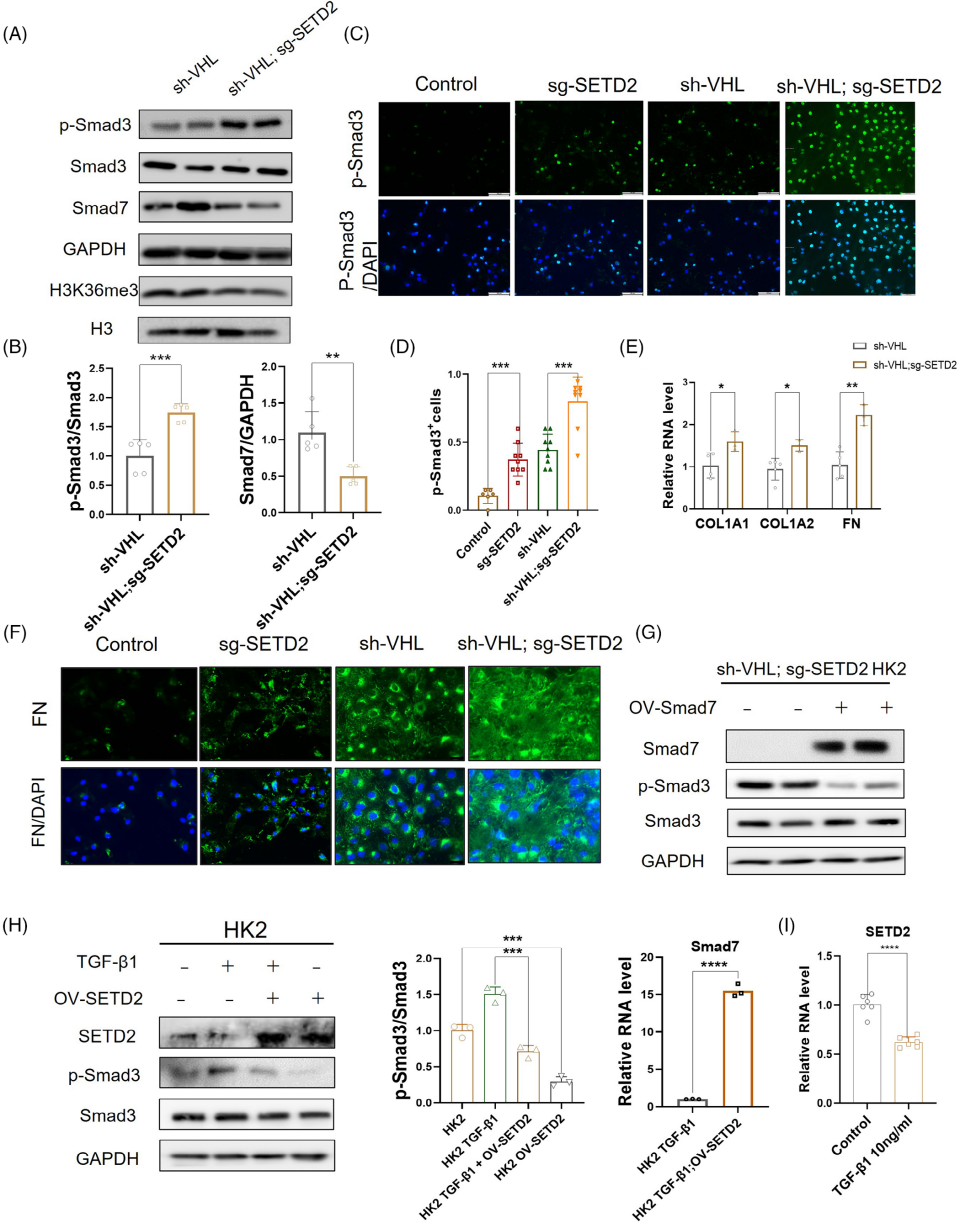
SET domain‐containing 2 (SETD2) deletion leads to fibrosis through activation of the transforming growth factor‐β (TGF‐β)/Smad signalling pathway in vitro. (A and B) The expression of p‐Smad3 and Smad7 in sh‐VHL and sh‐VHL; sg‐SETD2 HK2 cells (*n* = 4). (C) Immunofluorescence (IF) results of p‐Smad3 expression level in wild type (WT), sg‐SETD2, sh‐VHL and sh‐VHL; sg‐SETD2 HK2 cells (*n* = 4). Scale bars: 100 μm. (D) Statistical results of p‐Smad3 in WT, sg‐SETD2, sh‐VHL and sh‐VHL; sg‐SETD2 HK2 cells. (E) The mRNA levels of collagen type I alpha 1 chain (COL1A1), COL1A2 and fibronectin (FN) in sh‐VHL and sh‐VHL; sg‐SETD2 HK2 cells (*n* = 4). (F) IF results of FN expression level in WT, sg‐SETD2, sh‐VHL and sh‐VHL; sg‐SETD2 HK2 cells (*n* = 4). Scale bars: 100 μm. (G) Western blotting (WB) analysis of p‐Smad3 expression in sh‐VHL; sg‐SETD2 HK2 cells with or without OV‐Smad7. (H) WB analysis of p‐Smad3 expression in sh‐VHL; sg‐SETD2 HK2 cells with or without TGF‐β1 stimulation. (I) Changes in SETD2 mRNA expression level after TGF‐β1 stimulation. The data are represented as the mean ± standard deviation (SD). ^*^ *p* < .05, ^**^ *p* < .01, ^***^ *p* < .001, ^****^ *p* < .0001. VHL, Von Hippel–Lindau.

To further validate the effect of SETD2 on the TGF‐β/Smad signalling pathway and exclude the effect of the pro‐fibrotic environment, we deleted SETD2 through the CRISPR‐Cas9 method in HK2 cells. SETD2 deletion resulted in increased expression of p‐Smad3 and decreased expression of Smad7 in HK2 (Figure 6A,B). IF results presented that SETD2 deficiency resulted in the activation of the TGF‐β/Smad signalling pathway (Figure 6C,D). In addition, the mRNA levels of COL1A1, COL1A2 and FN1, which are the target genes of p‐Smad2/3, are increased in the absence of SETD2 (Figure 6E). IF results showed that FN protein expression levels increased after SETD2 deletion (Figure 6F). Considering that SETD2 inhibits TGF‐β/Smad signalling activation by regulating Smad7 expression, we performed a gene rescue experiment with Smad7 overexpression in HK2 cells. And the results showed that Smad7 overexpression inhibited the TGF‐β/Smad signal pathway caused by SETD2 and VHL deletion (Figure 6G). In addition, we further confirmed that SETD2 overexpression could facilitate Smad7 transcription to inhibit the TGF‐β/Smad signalling pathway and renal fibrosis (Figure 6H). We also found that TGF‐β can repress SETD2 expression (Figure 6I). These results indicated that SETD2 deletion leads to fibrogenesis through activation of the TGF‐β/Smad signalling pathway in vitro.

### 3.7 Treatment with TGF‐β/Smad inhibitor rescues the renal fibrosis phenotype caused by SETD2 absence

**FIGURE 7:**
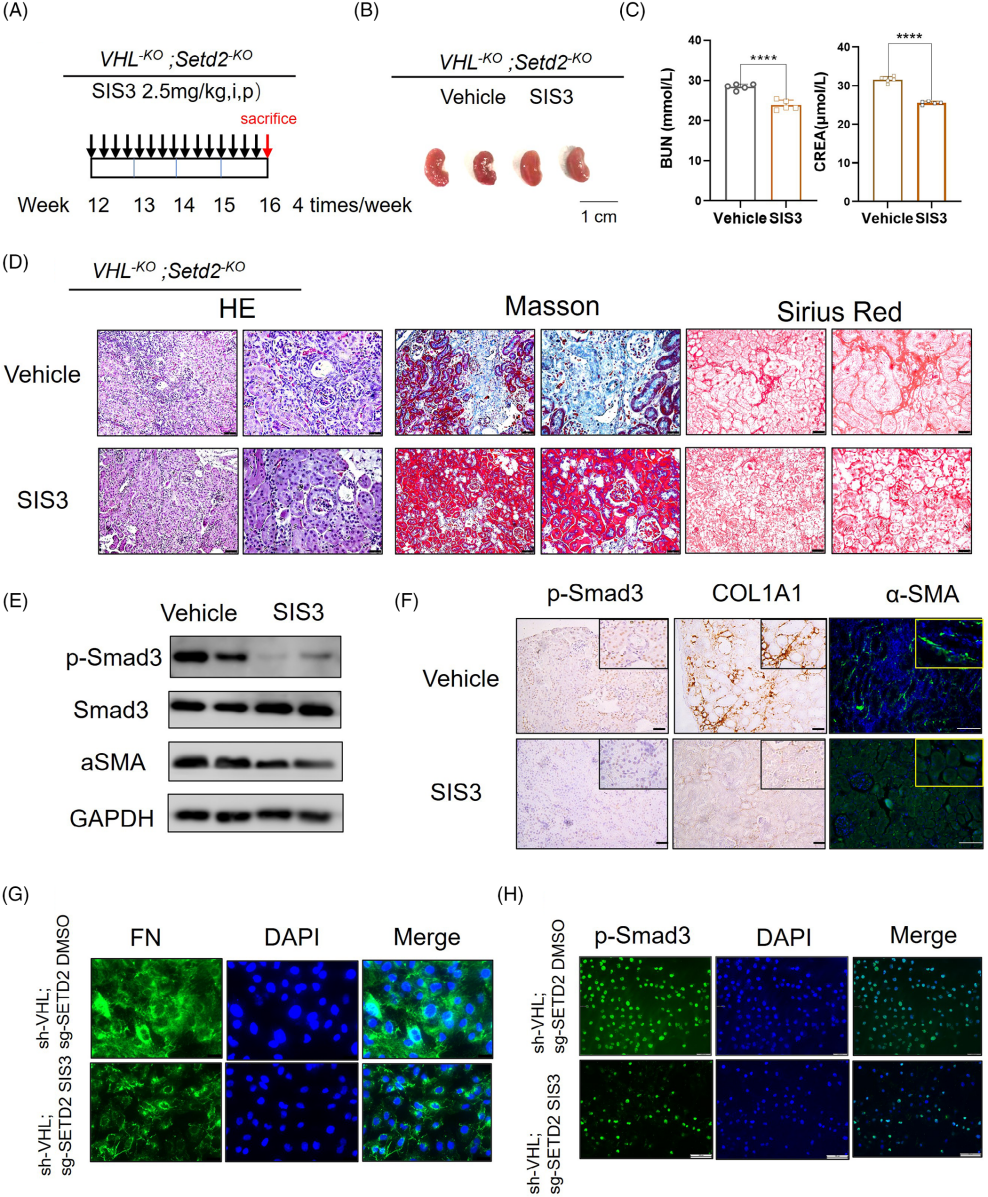
Treatment with transforming growth factor‐β (TGF‐β)/Smad inhibitor rescues the renal fibrosis phenotype caused by SET domain‐containing 2 (SETD2) absence. (A) A scheme of treatment was given for each injection (*n* = 5 mice per group). (B) Kidney volumes of *VHL^−KO^; Setd2^−KO^* mice treated with SIS3 or DMSO. Scale bars: 1 cm. (C) BUN and creatinine levels in *VHL^−KO^; Setd2^−KO^* mice treated with SIS3 or DMSO. (D) Haematoxylin and eosin (H&E), Masson's trichrome and Sirius Red staining and statistical results of *VHL^−KO^; Setd2^–KO^* mice treated with SIS3 or DMSO. (E) The protein levels of p‐Smad3 in *VHL^−KO^; Setd2^−KO^* mice treated with SIS3 or DMSO. (F) Immunohistochemistry (IHC) staining of p‐Smad3, collagen type I alpha 1 chain (COL1A1) and α‐smooth muscle actin (α‐SMA) in *VHL^−KO^; Setd2^−KO^* mice treated with SIS3 or DMSO. Scale bars: 50 μm. (G and H) Fibronectin (FN) and p‐Smad3 immunofluorescence (IF) results in sh‐VHL; sg‐SETD2 HK2 cells treated with SIS3 or DMSO. Scale bars: 100 μm. The data are represented as the mean ± standard deviation (SD). ^*^ *p* < .05, ^**^ *p* < .01, ^***^ *p* < .001, ^****^ *p* < .0001. VHL, Von Hippel–Lindau.

Given that SETD2 loss‐mediated fibrogenesis is dependent on the hyperactivation of TGF‐β/Smad signalling, we next investigated whether this phenotype could be rescued efficiently by the TGF‐β/Smad signalling inhibitor. SIS3 is a novel specific Smad3 inhibitor that inhibits TGF‐β by inhibiting Smad3 phosphorylation.^46^ SIS3 or DMSO was administered by i.p. injection four times a week for 4 weeks in 12‐week‐old *VHL^−KO^; Setd2^−KO^* mice. We found that the surface of SIS3‐treated mice was relatively smoother compared to the control group (Figure 7A,B). BUN and creatinine levels were reduced in the blood of SIS3‐treated mice compared to the control group (Figure 7C). In addition, the degree of kidney fibrosis was greatly reduced in SIS3‐treated mice compared to the control group (Figure 7D,E). Meanwhile, p‐Smad3, COL1A1 and α‐SMA positive staining were reduced in drug‐treated mice compared with controls, demonstrating that the TGF‐β/Smad inhibitor can largely relieve the fibrotic phenotype (Figure 7F,G). The same results were observed in vitro (Figure 7H,I). Overall, these results suggested that inhibition of the TGF‐β/Smad signalling pathway can rescue the renal fibrosis phenotype caused by SETD2 absence.

## 4 DISCUSSION

In this study, we demonstrated that SETD2 plays a critical role in the inhibition of the development of kidney fibrosis both in vitro and in vivo. We illustrated that SETD2 deficiency promotes renal fibrosis in VHL‐deficient mice by the activation of the TGF‐β/Smad signalling pathway. SETD2 maintains the expression level of Smad7 through H3K36me3 and inhibits the TGF‐β/Smad signalling pathway. The anti‐fibrotic effect of SETD2 provides an innovative insight into SETD2 as a potential therapeutic target for the treatment of renal fibrosis.

SETD2 is reported to be the only histone H3K36 trimethyltransferase involved in different biological processes, including DNA repair, transcription initiation and elongation^20^, ^21^, ^22^ SETD2‐mediated epigenetic alterations are associated with many diseases, especially in RCC.^23^, ^24^, ^25^ SETD2 loss perturbs the kidney cancer epigenetic landscape to promote metastasis and engenders actionable dependencies on histone chaperone complexes.^25^ Our recent studies reported that SETD2 plays important roles in intestinal immunity,^27^ suppressing inflammatory bowel disease,^28^ V(D)J recombination in normal lymphocyte development,^29^ attenuating experimental colitis,^30^ inhibiting pancreatic carcinogenesis,^31^ bone marrow mesenchymal stem cell differentiation,^32^ genomic imprinting, embryonic development,^33^ spermiogenesis^34^ and cutaneous wound healing.^35^ Based on the PKD model caused by the oncogene MYC, SETD2 deficiency accelerates the transition from PKD to RCC by regulating β‐catenin activity at the transcriptional and post‐transcriptional levels.^26^ In addition to the role of transcription regulation, SETD2 is also involved in non‐histone methylation modifications,^47^, ^48^ alternative splicing,^49^ etc. For example, it has been reported that SETD2 can restrict prostate cancer metastasis by integrating EZH2 and AMPK signalling pathways.^50^ Loss of SETD2 promotes acinar‐to‐ductal metaplasia and epithelial–mesenchymal transition during pancreatic carcinogenesis induced by Kras.^31^

There is evidence suggesting increased expression and activation of TGF‐β/Smad in human kidney disease. TGF‐β/Smad signalling pathway has important regulatory roles in inflammation, cancer, immunity and fibrosis.^51^, ^52^, ^53^ TGF‐β/Smad signalling pathway is the primary factor that drives renal fibrosis.^12^ Few studies have been conducted between SETD2 and the TGF‐β/Smad signalling pathway, especially in the field of renal diseases. Smad7 is a negative feedback inhibitor of the TGF‐β/Smad signalling pathway.^54^ Smad7 can recruit Smurf1 and Smurf2 to degrade TGFR1. In addition, Smad7 can inhibit the TGF‐β/Smad signalling pathway by competing with Smad2/3 for bounding to TGFR1 to prevent the phosphorylation of Smad2/3. SIS3 is a selective Smad3 inhibitor that attenuates TGF‐β1‐dependent Smad3 phosphorylation and DNA binding.^46^ SIS3 can alleviate many diseases, such as lung cancer.^55^ The results that SIS3 can treat fibrosis caused by SETD2 inactivation to some extent prove that the clinical application of SIS3 will be more desirable for patients with reduced SETD2 expression.

SETD2 and VHL are both commonly mutated genes in renal cell cancer. The expression of SETD2 and VHL is positively correlated in patients with renal fibrosis and renal cancer, indicating their clinical significance. Available studies have suggested that VHL protein can also ubiquitinate Smad3.^56^, ^57^ In this study, we found that SETD2 and VHL could jointly inhibit TGF‐β/Smad signalling pathway activation. Deletion of SETD2 results in reduced expression of Smad7, leading to the failure to inhibit the TGF‐β signalling pathway. Meanwhile, the lack of VHL leads to the excessive accumulation of Smad3, which promotes the TGF‐β signalling pathway. The combined effect of the two aspects results in the excessive activation of the TGF‐β signalling pathway. However, as important regulatory molecules, SETD2 and VHL are involved in various biological processes. The potential interaction between VHL and SETD2 is unknown and undoubtedly complex. And it will also be a major focus of our future work.

In summary, we established a mouse model of renal fibrosis driven by deficiency of SETD2 and VHL. SETD2 deficiency resulted in severe renal fibrosis in the absence of VHL, and based on these results, we found that SETD2 deletion can lead to reduced expression of Smad7 by H3K36me3, which activates the TGF‐β/Smad signalling pathway and leads to renal fibrosis. In addition, TGF‐β inhibitor can rescue the phenotype caused by SETD2 absence. For clinical translation, pharmaceutical investigation of the cross‐talk between SETD2 and TGF‐β/Smad signalling may provide a potentially promising strategy to prevent renal fibrogenesis in patients (Supporting Information).

## CONFLICT OF INTEREST STATEMENT

The authors declare they have no conflicts of interest.

## Supporting information

Supporting Information

## Data Availability

The datasets used and/or analysed during this study are available from the corresponding author upon reasonable request. RNA sequencing raw data have been deposited in the Gene Expression Omnibus (GEO) under accession number GEO: GSE213922. ChIP sequencing: GSE125528.

## References

[1] Kalantar‐Zadeh K , Jafar TH , Nitsch D , Neuen BL , Perkovic V . Chronic kidney disease. Lancet North Am Ed. 2021;398(10302):786‐802. doi:10.1016/S0140-6736(21)00519-5 34175022

[2] Romagnani P , Remuzzi G , Glassock R , et al. Chronic kidney disease. Nat Rev Dis Primers. 2017;3(1):17088. doi:10.1038/nrdp.2017.88 29168475

[3] Liu Y . Renal fibrosis: new insights into the pathogenesis and therapeutics. Kidney Int. 2006;69(2):213‐217. doi:10.1038/sj.ki.5000054 16408108

[4] Bello AK , Levin A , Lunney M , et al. Status of care for end stage kidney disease in countries and regions worldwide: international cross sectional survey. BMJ. 2019;367:l5873. doi:10.1136/bmj.l5873 31672760

[5] Okuda Y , Soohoo M , Tang Y , et al. Estimated GFR at dialysis initiation and mortality in children and adolescents. Am J Kidney Dis. 2019;73(6):797‐805. doi:10.1053/j.ajkd.2018.12.038 30833086

[6] Kurella Tamura M , Desai M , Kapphahn KI , Thomas IC , Asch SM , Chertow GM . Dialysis versus medical management at different ages and levels of kidney function in veterans with advanced CKD. JASN. 2018;29(8):2169‐2177. doi:10.1681/ASN.2017121273 29789430 PMC6065074

[7] Crews DC , Scialla JJ , Boulware LE , et al. Comparative effectiveness of early versus conventional timing of dialysis initiation in advanced CKD. Am J Kidney Dis. 2014;63(5):806‐815. doi:10.1053/j.ajkd.2013.12.010 24508475 PMC4117406

[8] Yamamoto T , Nakamura T , Noble NA , Ruoslahti E , Border WA . Expression of transforming growth factor 18 is elevated in human and experimental diabetic nephropathy. Med Sci. 1993:5. 10.1073/pnas.90.5.1814 PMC45970 7680480

[9] Wang SN , Lapage J , Hirschberg R . Role of glomerular ultrafiltration of growth factors in progressive interstitial fibrosis in diabetic nephropathy. Kidney Int. 2000;57(3):1002‐1014. doi:10.1046/j.1523-1755.2000.00928.x 10720953

[10] Sato M , Muragaki Y , Saika S , Roberts AB , Ooshima A . Targeted disruption of TGF‐β1/Smad3 signaling protects against renal tubulointerstitial fibrosis induced by unilateral ureteral obstruction. J Clin Invest. 2003;112(10):1486‐1494. doi:10.1172/JCI200319270 14617750 PMC259132

[11] Inazaki K , Kanamaru Y , Kojima Y , et al. Smad3 deficiency attenuates renal fibrosis, inflammation, and apoptosis after unilateral ureteral obstruction. Kidney Int. 2004;66(2):597‐604. doi:10.1111/j.1523-1755.2004.00779.x 15253712

[12] Meng X , Nikolic‐Paterson DJ , Lan HY . TGF‐β: the master regulator of fibrosis. Nat Rev Nephrol. 2016;12(6):325‐338. doi:10.1038/nrneph.2016.48 27108839

[13] Kavsak P , Rasmussen RK , Causing CG , et al. Smad7 binds to Smurf2 to form an E3 ubiquitin ligase that targets the TGFβ receptor for degradation. Mol Cell. 2000;6(6):1365‐1375. 11163210 10.1016/s1097-2765(00)00134-9

[14] Shi Y , Massagué J . Mechanisms of TGF‐beta signaling from cell membrane to the nucleus. Cell. 2003;113(6):685‐700. doi:10.1016/s0092-8674(03)00432-x 12809600

[15] Kato M , Natarajan R . Diabetic nephropathy–emerging epigenetic mechanisms. Nat Rev Nephrol. 2014;10(9):517‐530. doi:10.1038/nrneph.2014.116 25003613 PMC6089507

[16] Skvortsova K , Iovino N , Bogdanović O . Functions and mechanisms of epigenetic inheritance in animals. Nat Rev Mol Cell Biol. 2018;19(12):774‐790. doi:10.1038/s41580-018-0074-2 30425324

[17] Majumder S , Thieme K , Batchu SN , et al. Shifts in podocyte histone H3K27me3 regulate mouse and human glomerular disease. J Clin Invest. 2017;128(1):483‐499. doi:10.1172/JCI95946 29227285 PMC5749498

[18] Sun G , Reddy MA , Yuan H , Lanting L , Kato M , Natarajan R . Epigenetic histone methylation modulates fibrotic gene expression. J Am Soc Nephrol. 2010;21(12):2069‐2080. doi:10.1681/ASN.2010060633 20930066 PMC3014020

[19] Edmunds JW , Mahadevan LC , Clayton AL . Dynamic histone H3 methylation during gene induction: HYPB/Setd2 mediates all H3K36 trimethylation. EMBO J. 2008;27(2):406‐420. doi:10.1038/sj.emboj.7601967 18157086 PMC2168397

[20] Kanu N , Grönroos E , Martinez P , et al. SETD2 loss‐of‐function promotes renal cancer branched evolution through replication stress and impaired DNA repair. Oncogene. 2015;34(46):5699‐5708. doi:10.1038/onc.2015.24 25728682 PMC4660036

[21] Zhang Y , Xie S , Zhou Y , et al. H3K36 histone methyltransferase Setd2 is required for murine embryonic stem cell differentiation toward endoderm. Cell Rep. 2014;8(6):1989‐2002. doi:10.1016/j.celrep.2014.08.031 25242323

[22] Yuan H , Li N , Fu D , et al. Histone methyltransferase SETD2 modulates alternative splicing to inhibit intestinal tumorigenesis. J Clin Invest. 2017;127(9):3375‐3391. doi:10.1172/JCI94292 28825595 PMC5669571

[23] Gerlinger M , Horswell S , Larkin J , et al. Genomic architecture and evolution of clear cell renal cell carcinomas defined by multiregion sequencing. Nat Genet. 2014;46(3):225‐233. doi:10.1038/ng.2891 24487277 PMC4636053

[24] Morris MR , Latif F . The epigenetic landscape of renal cancer. Nat Rev Nephrol. 2017;13(1):47‐60. doi:10.1038/nrneph.2016.168 27890923

[25] Xie Y , Sahin M , Sinha S , et al. SETD2 loss perturbs the kidney cancer epigenetic landscape to promote metastasis and engenders actionable dependencies on histone chaperone complexes. Nat Cancer. 2022;3(2):188‐202. doi:10.1038/s43018-021-00316-3 35115713 PMC8885846

[26] Rao H , Li X , Liu M , et al. Multilevel regulation of β‐catenin activity by SETD2 suppresses the transition from polycystic kidney disease to clear cell renal cell carcinoma. Cancer Res. 2021;81(13):3554‐3567. doi:10.1158/0008-5472.CAN-20-3960 33910928

[27] Chang J , Ji X , Deng T , et al. Setd2 determines distinct properties of intestinal ILC3 subsets to regulate intestinal immunity. Cell Rep. 2022;38(11):110530. doi:10.1016/j.celrep.2022.110530 35294891

[28] Chen Y , Liu M , Wang W , et al. Loss of Setd2 associates with aberrant microRNA expression and contributes to inflammatory bowel disease progression in mice. Genomics. 2021;113(4):2441‐2454. doi:10.1016/j.ygeno.2021.05.034 34052319

[29] Ji Z , Sheng Y , Miao J , et al. The histone methyltransferase Setd2 is indispensable for V(D)J recombination. Nat Commun. 2019;10(1):3353. doi:10.1038/s41467-019-11282-x 31350389 PMC6659703

[30] Liu M , Rao H , Liu J , et al. The histone methyltransferase SETD2 modulates oxidative stress to attenuate experimental colitis. Redox Biol. 2021;43:102004. doi:10.1016/j.redox.2021.102004 34020310 PMC8141928

[31] Niu N , Lu P , Yang Y , et al. Loss of Setd2 promotes Kras‐induced acinar‐to‐ductal metaplasia and epithelia–mesenchymal transition during pancreatic carcinogenesis. Gut. 2020;69(4):715‐726. doi:10.1136/gutjnl-2019-318362 31300513

[32] Wang L , Niu N , Li L , Shao R , Ouyang H , Zou W . H3K36 trimethylation mediated by SETD2 regulates the fate of bone marrow mesenchymal stem cells. PLoS Biol. 2018;16(11):e2006522. doi:10.1371/journal.pbio.2006522 30422989 PMC6233919

[33] Xu Q , Xiang Y , Wang Q , et al. SETD2 regulates the maternal epigenome, genomic imprinting and embryonic development. Nat Genet. 2019;51(5):844‐856. doi:10.1038/s41588-019-0398-7 31040401

[34] Zuo X , Rong B , Li L , Lv R , Lan F , Tong MH . The histone methyltransferase SETD2 is required for expression of acrosin‐binding protein 1 and protamines and essential for spermiogenesis in mice. J Biol Chem. 2018;293(24):9188‐9197. doi:10.1074/jbc.RA118.002851 29716999 PMC6005419

[35] Li X , Liu C , Zhu Y , et al. SETD2 epidermal deficiency promotes cutaneous wound healing via activation of AKT/mTOR signalling. Cell Prolif. 2021;54(6):e13045. doi:10.1111/cpr.13045 33949020 PMC8168411

[36] Dizman N , Philip EJ , Pal SK . Genomic profiling in renal cell carcinoma. Nat Rev Nephrol. 2020;16(8):435‐451. doi:10.1038/s41581-020-0301-x 32561872

[37] Gossage L , Eisen T , Maher ER . VHL, the story of a tumour suppressor gene. Nat Rev Cancer. 2015;15(1):55‐64. doi:10.1038/nrc3844 25533676

[38] Haase VH . The VHL/HIF oxygen‐sensing pathway and its relevance to kidney disease. Kidney Int. 2006;69(8):1302‐1307. doi:10.1038/sj.ki.5000221 16531988

[39] Lehmann H , Vicari D , Wild PJ , Frew IJ . Combined deletion of Vhl and Kif3a accelerates renal cyst formation. J Am Soc Nephrol. 2015;26(11):2778. doi:10.1681/ASN.2014090875 25788526 PMC4625677

[40] Guinot A , Lehmann H , Wild PJ , Frew IJ . Combined deletion of Vhl, Trp53 and Kif3a causes cystic and neoplastic renal lesions. J Pathol. 2016;239(3):365‐373. doi:10.1002/path.4736 27126173

[41] Albers J , Rajski M , Schönenberger D , et al. Combined mutation of Vhl and Trp53 causes renal cysts and tumours in mice. EMBO Mol Med. 2013;5(6):949‐964. doi:10.1002/emmm.201202231 23606570 PMC3779454

[42] Terryn S , Jouret F , Vandenabeele F , et al. A primary culture of mouse proximal tubular cells, established on collagen‐coated membranes. Am J Physiol Renal Physiol. 2007;293(2):F476‐485. doi:10.1152/ajprenal.00363.2006 17475898

[43] Wang Y , Wang M , Ning F , et al. A novel role of BK potassium channel activity in preventing the development of kidney fibrosis. Kidney Int. 2022;101(5):945‐962. doi:10.1016/j.kint.2021.11.033 34968553

[44] Chen YT , Jhao PY , Hung CT , et al. Endoplasmic reticulum protein TXNDC5 promotes renal fibrosis by enforcing TGF‐β signaling in kidney fibroblasts. J Clin Invest. 2021;131(5):e143645. doi:10.1172/JCI143645 33465051 PMC7919722

[45] Nguyen‐Tran HH , Nguyen TN , Chen CY , Hsu T . Endothelial reprogramming stimulated by oncostatin M promotes inflammation and tumorigenesis in VHL‐deficient kidney tissue. Cancer Res. 2021;81(19):5060‐5073. doi:10.1158/0008-5472.CAN-21-0345 34301760 PMC8974431

[46] Jinnin M , Ihn H , Tamaki K . Characterization of SIS3, a novel specific inhibitor of Smad3, and its effect on transforming growth factor‐beta1‐induced extracellular matrix expression. Mol Pharmacol. 2006;69(2):597‐607. doi:10.1124/mol.105.017483 16288083

[47] Park IY , Powell RT , Tripathi DN , et al. Dual chromatin and cytoskeletal remodeling by SETD2. Cell. 2016;166(4):950‐962. doi:10.1016/j.cell.2016.07.005 27518565 PMC5101839

[48] Chen K , Liu J , Liu S , et al. Methyltransferase SETD2‐mediated methylation of STAT1 is critical for interferon antiviral activity. Cell. 2017;170(3):492‐506.e14. doi:10.1016/j.cell.2017.06.042 28753426

[49] Kornblihtt AR . Epigenetics at the base of alternative splicing changes that promote colorectal cancer. J Clin Invest. 2017;127(9):3281‐3283. doi:10.1172/JCI96497 28825597 PMC5669579

[50] Yuan H , Han Y , Wang X , et al. SETD2 restricts prostate cancer metastasis by integrating EZH2 and AMPK signaling pathways. Cancer Cell. 2020;38(3):350‐365. doi:10.1016/j.ccell.2020.05.022 32619406

[51] Batlle E , Massagué J . Transforming growth factor‐β signaling in immunity and cancer. Immunity. 2019;50(4):924‐940. doi:10.1016/j.immuni.2019.03.024 30995507 PMC7507121

[52] Lan HY . Diverse roles of TGF‐β/Smads in renal fibrosis and inflammation. Int J Biol Sci. 2011;7(7):1056‐1067. doi:10.7150/ijbs.7.1056 21927575 PMC3174390

[53] Nolte M , Margadant C . Controlling immunity and inflammation through integrin‐dependent regulation of TGF‐β. Trends Cell Biol. 2020;30(1):49‐59. doi:10.1016/j.tcb.2019.10.002 31744661

[54] Monteleone G , Pallone F , MacDonald TT . Smad7 in TGF‐beta‐mediated negative regulation of gut inflammation. Trends Immunol. 2004;25(10):513‐517. doi:10.1016/j.it.2004.07.008 15364052

[55] Lian GY , Wan Y , Mak TSK , et al. Self‐carried nanodrug (SCND‐SIS3): a targeted therapy for lung cancer with superior biocompatibility and immune boosting effects. Biomaterials. 2022;288:121730. doi:10.1016/j.biomaterials.2022.121730 35995622

[56] Zhou J , Dabiri Y , Gama‐Brambila RA , et al. pVHL‐mediated SMAD3 degradation suppresses TGF‐β signaling. J Cell Biol. 2021;221(1):e202012097. doi:10.1083/jcb.202012097 34860252 PMC8650352

[57] Yang J , Ruan Y , Wang D , et al. VHL‐recruiting PROTAC attenuates renal fibrosis and preserves renal function via simultaneous degradation of Smad3 and stabilization of HIF‐2α. Cell Biosci. 2022;12(1):203. doi:10.1186/s13578-022-00936-x 36536448 PMC9761961

