# Supplementary material for: SETD2 deficiency promotes renal fibrosis through the TGF‐β/Smad signalling pathway in the absence of VHL
Source: Clin Transl Med. 2023 Nov 7;13(11):e1468. doi: 10.1002/ctm2.1468 (PMC10629155; doi:10.1002/ctm2.1468)
Supplement: Supplementary file 1 — Supporting Information [file CTM2-13-e1468-s001.docx]

**Supplementary material**

**Primers**

Primers for Real-time PCR and ChIP-qPCR

| mTgfbr1-F | TCTGCATTGCACTTATGCTGA |
| --- | --- |
| mTgfbr1-R | AAAGGGCGATCTAGTGATGGA |
| mTgfb2-F | CTTCGACGTGACAGACGCT |
| mTgfb2-R | GCAGGGGCAGTGTAAACTTATT |
| msmad4-F | ACACCAACAAGTAACGATGCC |
| msmad4-R | GCAAAGGTTTCACTTTCCCCA |
| mCOL1A1-F | GCTCCTCTTAGGGGCCACT |
| mCOL1A1-R | CCACGTCTCACCATTGGGG |
| mFibronectin-F | ATGTGGACCCCTCCTGATAGT |
| mFibronectin-R | GCCCAGTGATTTCAGCAAAGG |
| mαSMA-F | GTCCCAGACATCAGGGAGTAA |
| mαSMA-R | TCGGATACTTCAGCGTCAGGA |
| mTNFα-F | CGTCAGCCGATTTGCTATCT |
| mTNFα-R | CGGACTCCGCAAAGTCTAAG |
| mIL-6-F | AGTTGCCTTCTTGGGACTGA |
| mIL-6-R | CAGAATTGCCATTGCACAAC |
| mIL-1α-F | GAGAGCCGGGTGACAGTATC |
| mIL-1α-R | TGACAAACTTCTGCCTGACG |
| mIL-1β-F | GAAATGCCACCTTTTGACAGTG |
| mIL-1β-R | TGGATGCTCTCATCAGGACAG |
| mIL-13-F | AAGGAGCTTATTGAGGAGCTG |
| mIL-13-R | TCAGGGAATCCAGGGCTACA |
| mCcl2-F | TTAAAAACCTGGATCGGAACCAA |
| mCcl2-R | GCATTAGCTTCAGATTTACGGGT |
| mCxcl1-F | CTGGGATTCACCTCAAGAACATC |
| mCxcl1-R | CAGGGTCAAGGCAAGCCTC |
| mGapdh-F | AGGTCGGTGTGAACGGATTTG |
| mGapdh-R | TGTAGACCATGTAGTTGAGGTCA |
| hGapdh-F | AGAAGGCTGGGGCTCATTTG |
| hGapdh-R | AGGGGCCATCCACAGTCTTC |
| mSmad7-F | GGCCGGATCTCAGGCATTC |
| mSmad7-R | TTGGGTATCTGGAGTAAGGAGG |
| hSmad7-F | GGACAGCTCAATTCGGACAAC |
| hSamd7-R | GTACACCCACACACCATCCAC |
| (ChIP)Smad7-1 F | CGCCTGCTGCCCCAACTCGGCG |
| (ChIP)Smad7-1 R | GTCGTTTGCCGGCTAAGGAGCG |
| (ChIP)Smad7-2 F | CAGTCCTAGGGCCCATCCCT |
| (ChIP)Smad7-2 R | AGGACTTGTCCCTGCGGCC |
| (ChIP)Smad7-4 F | GGTGGCATACTGGGAGGAG |
| (ChIP)Smad7-4 R | GATATCCAGGGAGGGCTCTTG |
| mSETD2-F | CATAGCTGTGAACCAAACTGTGA |
| mSETD2-R | TAATTCTGAGCCTGAAGGAACTA |
| hSETD2-F | GAACCCTTACCGGAAACCTGA |
| hSETD2-R | CAGGTCCTCAGGATTCTTACAG |

Primers for CRISPR/Cas9 mediated SETD2 knockout and shRNA vector construction

| sg-SETD2 Sense 5’-3’ | caccgAGAAATCAGTCGGCAGGACA |
| --- | --- |
| sg-SETD2 Antisense 5’-3’ | aaacTGTCCTGCCGACTGATTTCTC |
| sh-VHL | GCCTGAGAATTACAGGAGACTCGAGTCTCCTGTAATTCTC AGGC |
